# Ebola Virus RNA Editing Depends on the Primary Editing Site Sequence and an Upstream Secondary Structure

**DOI:** 10.1371/journal.ppat.1003677

**Published:** 2013-10-17

**Authors:** Masfique Mehedi, Thomas Hoenen, Shelly Robertson, Stacy Ricklefs, Michael A. Dolan, Travis Taylor, Darryl Falzarano, Hideki Ebihara, Stephen F. Porcella, Heinz Feldmann

**Affiliations:** 1 Department of Medical Microbiology, University of Manitoba, Winnipeg, Manitoba, Canada; 2 Laboratory of Virology, Rocky Mountain Laboratories, Division of Intramural Research, National Institute of Allergy and Infectious Diseases, National Institutes of Health, Hamilton, Montana, United States of America; 3 Research Technology Branch, Genomics Unit, Rocky Mountain Laboratories, National Institute of Allergy and Infectious Diseases, National Institutes of Health, Hamilton, Montana, United States of America; 4 Bioinformatics and Computational Bioscience Branch, National Institute of Allergy and Infectious Diseases, National Institutes of Health, Bethesda, Maryland, United States of America; The Scripps Research Institute, United States of America

## Abstract

Ebolavirus (EBOV), the causative agent of a severe hemorrhagic fever and a biosafety level 4 pathogen, increases its genome coding capacity by producing multiple transcripts encoding for structural and nonstructural glycoproteins from a single gene. This is achieved through RNA editing, during which non-template adenosine residues are incorporated into the EBOV mRNAs at an editing site encoding for 7 adenosine residues. However, the mechanism of EBOV RNA editing is currently not understood. In this study, we report for the first time that minigenomes containing the glycoprotein gene editing site can undergo RNA editing, thereby eliminating the requirement for a biosafety level 4 laboratory to study EBOV RNA editing. Using a newly developed dual-reporter minigenome, we have characterized the mechanism of EBOV RNA editing, and have identified cis-acting sequences that are required for editing, located between 9 nt upstream and 9 nt downstream of the editing site. Moreover, we show that a secondary structure in the upstream cis-acting sequence plays an important role in RNA editing. EBOV RNA editing is glycoprotein gene-specific, as a stretch encoding for 7 adenosine residues located in the viral polymerase gene did not serve as an editing site, most likely due to an absence of the necessary cis-acting sequences. Finally, the EBOV protein VP30 was identified as a trans-acting factor for RNA editing, constituting a novel function for this protein. Overall, our results provide novel insights into the RNA editing mechanism of EBOV, further understanding of which might result in novel intervention strategies against this viral pathogen.

## Author Summary

Ebola virus (EBOV) causes severe hemorrhagic fever with case fatality rates of up to 90% and no therapy or vaccine currently available. A better understanding of the EBOV life cycle is important to develop new countermeasures against this virus; however, research with live EBOV is restricted to high containment laboratories. One unique feature of the EBOV life cycle is that its surface glycoprotein is expressed only after editing of the glycoprotein mRNA by the viral polymerase, leading to an insertion of a non-templated nucleotide into the mRNA. While this phenomenon has been long known, the mechanism of mRNA editing for EBOV is not understood. We have developed a unique minigenome system that allows the study of EBOV mRNA editing outside of a high containment laboratory. Using this system we have characterized EBOV mRNA editing and defined the sequence requirements for this process. Interestingly, we could show that signals both up- and downstream of the editing site are important, and that a secondary structure in the RNA upstream of the editing site as well as the viral protein VP30 contribute to editing. These findings provide new detailed molecular information about an essential process in the EBOV life cycle, which might be a potential novel target for antivirals.

## Introduction

Filoviruses (ebolaviruses (EBOV) and marburgviruses (MARV)) cause severe hemorrhagic fever in humans and nonhuman primates [1]. They contain a non-segmented negative-sense single-stranded RNA genome accommodating seven genes (NP, VP35, VP40, GP, VP30, VP24, and L) to produce seven structural proteins (nucleoprotein, polymerase cofactor, major matrix protein, transmembrane glycoprotein, transcription activator, minor matrix protein, and RNA dependent RNA polymerase, respectively) [2]. The transmembrane glycoprotein (GP_1,2_) plays an important role in pathogenesis by dictating viral tissue tropism and initiating infection [2]. Despite similarities in genome and protein functions between EBOV and MARV, one of the major differences is that only EBOV increases its genome coding capacity by producing multiple transcripts from the GP gene using RNA editing [2], [3]. The EBOV ribonucleoprotein (RNP) complex, consisting of NP, VP35, L, and VP30, edits the GP gene at an editing site (seven consecutive uridine (U) residues in the genomic vRNA) by introducing non-template adenosine residues into the mRNA to produce multiple transcript species [3]–[5]. Unedited transcripts (seven adenosine residues at the editing site) of the GP gene encode for a soluble form of the glycoprotein (sGP). In contrast, edited transcripts in which an eighth or ninth adenosine residue is inserted at the editing site, resulting in a +1- or +2-shift in the open reading frame (ORF), encode GP_1,2_ and the small soluble glycoprotein (ssGP) [3]–[5]. A knockout of the editing site in a recombinant *Zaire ebolavirus* (ZEBOV) resulted in a significant increase in cytopathogenicity compared to wild-type virus, indicating the importance of RNA editing for regulating GP_1,2_ expression and reducing early cytotoxicity during EBOV infection [6], [7]. Despite this and potential other unknown functions of RNA editing, the mechanism of EBOV RNA editing has not yet been characterized. In particular, it is unknown what regions in the GP gene sequence are required, and whether any viral trans-acting factors contribute to RNA editing.

As a first-step to characterize RNA editing we utilized ZEBOV minigenome systems, which allowed us to study viral transcription and replication under biosafety level (BSL) 2 conditions [8], [9]. In particular, we developed a dual-reporter minigenome, with which we were able to show that the conserved editing site in the GP gene as well as neighboring sequences are essential for editing. In addition, VP30 was identified as a trans-acting factor for RNA editing. Finally, we could show that EBOV RNA editing is GP gene-specific, because a sequence located in L gene encoding for seven consecutive adenosine residues did not serve as an editing site, most likely due to the lack of necessary cis-acting sequences.

## Materials and Methods

### Cloning and mutagenesis

Minigenomes containing the entire coding region or parts of the coding region of the GP and parts of the coding region of the L gene were inserted into the published ZEBOV minigenome plasmid [8] by replacing the previously used reporter gene using standard cloning techniques. To generate the dual-reporter cassette, the ORFs for the enhanced green fluorescent protein (eGFP) and mCherry were cloned up- and downstream, respectively, of 110 nt of the GP gene surrounding the editing site, with the mCherry ORF shifted in a way that functional expression of mCherry would require insertion of an additional residue into the editing site of the mRNA. Subsequently, the dual-reporter cassette was cloned into the minigenome plasmid, as well as into pCAGGS (mammalian expression vector) and pTM1 (T7-driven expression vector) for control experiments. An altered version of the dual-reporter cassette containing an additional A residue in the editing site for control experiments was generated and cloned into pCAGGS. Point mutations and deletions were introduced into the editing site and surrounding sequences using PCR-driven technology. Two potential stem-loops were predicted in the 45 nt upstream of the editing site using the RNA secondary structure prediction Mfold webserver [10]. The XRNAmute webserver [11] was used to identify point mutations destabilizing these stem-loops, which were introduced into the minigenome plasmid using site directed mutagenesis. All plasmids were sequence verified. Primer sequences and detailed cloning strategies are provided in the supplementary information (Text S1).

### Cell culture and minigenome assays

293T (human embryonic kidney cell line) cells were cultured in Dulbecco's minimal essential medium (DMEM) (Sigma-Aldrich) supplemented with 10% (v/v) fetal bovine serum (FBS) (heat inactivated) (Invitrogen), 1% L-glutamine (2 mM) (Gibco) and 1% penicillin/streptomycin (100 U/ml) (Gibco) under 5% CO_2_ in a humidified incubator at 37°C. For minigenome rescues, 293T cells were seeded one day before transfection for 50–60% confluency at the time of transfection. Transfection was performed using TransIT-LT1 (Mirus) according to the manufacturer's instructions, and 250 ng minigenome plasmid, 1000 ng pCAGGS-L, 125 ng pCAGGS-VP35, 125 ng pCAGGS-NP, 75 ng pCAGGS-VP30, and 250 ng pCAGGS-T7. Cells were analyzed for reporter gene expression 48 hrs post transfection (unless otherwise stated).

### Transcript quantification

For quantification of transcripts, minigenome assays were performed as described above, but in vitro transcribed minigenome RNA was used instead of minigenome plasmid DNA to minimize the potential of plasmid contamination in the subsequent transcript quantification steps. Minigenome RNA was in vitro-transcribed using the MAXIscript T7 kit (Ambion), according to the manufacturer's instructions. Briefly, 2 ug minigenome plasmid DNA was linearized using SmaI, precipitated with ammonium acetate, and 1 ug DNA was then used for in vitro-transcription. After in-vitro transcription, residual plasmid DNA was removed using Turbo DNase (Ambion), and transcripts were precipitated with ammonium acetate. For RNA minigenome rescues, 293T cells were transfected as described above, but pCAGGS-T7 and the minigenome plasmids were omitted from the transfection mix. 24 hrs later RNA transfection was performed using the TransIT-mRNA transfection kit (Mirus) according to the manufacturer's instructions, using 1 ug of RNA transcript, 2.5 ul mRNA boost reagent and 2.5 ul mRNA TransIT-mRNA reagent. 48 hrs later, total RNA was extracted from the cells using the RNeasy Kit (Qiagen), including the optional DNAse I treatments, according to the manufacturer's instructions. Transcript quantification was done using a rapid transcript quantification assay (RTQA). Briefly, an oligo-dT based first-strand synthesis was performed on extracted RNA using SuperScript III (Invitrogen) according to the manufacturer's instructions. cDNA was purified and then subjected to PCR using a 6 carboxyfluorecein (FAM)-labeled forward primer, followed by capillary electrophoresis-based fragment length analysis using the Genetic Analyzer 3730xl (Applied Biosystems). PCR reactions were done in triplicates for each sample. Importantly, controls omitting reverse transcriptase showed the absence of DNA contaminations in our RNA preparations (data not shown).

### Detection and quantification of proteins

EBOV glycoproteins were detected following sodium dodecyl sulfate-polyacrylamide gel electrophoresis (SDS-PAGE) under reducing conditions using a rabbit anti-peptide antibody (peptide TIGEWAFWETKKPH; anti-ssGP/sGP/GP_1,2_) (1∶1,000 dilution), which is directed against the the first 12 amino acid residues common to ssGP, sGP, and GP_1,2_ and was purchased from Mimotopes. Dnk anti-Rabbit IgG (H+L) (Jackson ImmunoResearch) (1/10,000 dilution) was used as a secondary antibody and bound antibody was detected using the ECL Plus western blotting detection kit (GE Healthcare).

For confocal microscopy experiments, transfection was done in a six-well plate containing coverslips as described above, and 48 hrs after transfection coverslips were washed with phosphate buffered saline (PBS), fixed with 4% paraformaldehyde (PFA), and mounted using ProLong Gold Antifade reagent with 4′,6-diamidino-2-phenylindole (DAPI) (Invitrogen). Confocal images were obtained by a Zeiss LSM710 confocal microscope with a 63× oil immersion objective in sequential excitation mode. Slides were also analyzed using an AXIO Imager M1 epi-fluorescence microscope (Zeiss) where applicable.

For flow cytometry, cells were fixed with 2% PFA, and washed with cold (4°C) PBS containing 2% FBS. Cells were then analyzed on a LSR II Flow Cytometer (BD Biosciences). Cells were initially gated based on forward and side scatter to exclude dead cells and debris. Mean fluorescent intensity (MFI) of eGFP and mCherry was then measured on gated eGFP positive cells. At least 100,000 events were analyzed for each sample. Data analysis was performed using FlowJo software version 8.3.3 (TreeStar Inc.).

### Molecular dynamics

A three-dimensional model of the GGGAAACU stem-loop structure was constructed based on an NMR-derived structure of a tetraloop-receptor complex [12]. The model was explicitly solvated with TIP3P water molecules and Na+ and Cl- counterions using the VMD program [13]. Molecular dynamics simulations were performed under isobaric-isothermal conditions with periodic boundary conditions using the NAMD [14] program (v.2.7) on the Biowulf Linux cluster at the National Institutes of Health, Bethesda, MD (http://biowulf.nih.gov). Electrostatic interactions were calculated using the Particle-Mesh Ewald summation. The CHARMM27 [15] forcefield was used with CHARMM atom types and charges. Prior to the start of the simulation, an energy minimization was performed using a conjugate gradient method, followed by slow warming to 310 K in 10 K increments. Each increment ran for 5 psec in order to equilibrate the system at a given temperature. Production runs were conducted at 310 K for 18 nsec with data collected every nsec. For all simulations, a 1 fsec integration timestep was used along with a 12 Å non-bonded term cutoff. Langevin dynamics were used to maintain temperature and a modified Nosé-Hoover Langevin piston was used to control pressure.

## Results

### EBOV RNA editing can be modeled using minigenome systems and is GP gene-specific

**Figure 1:**
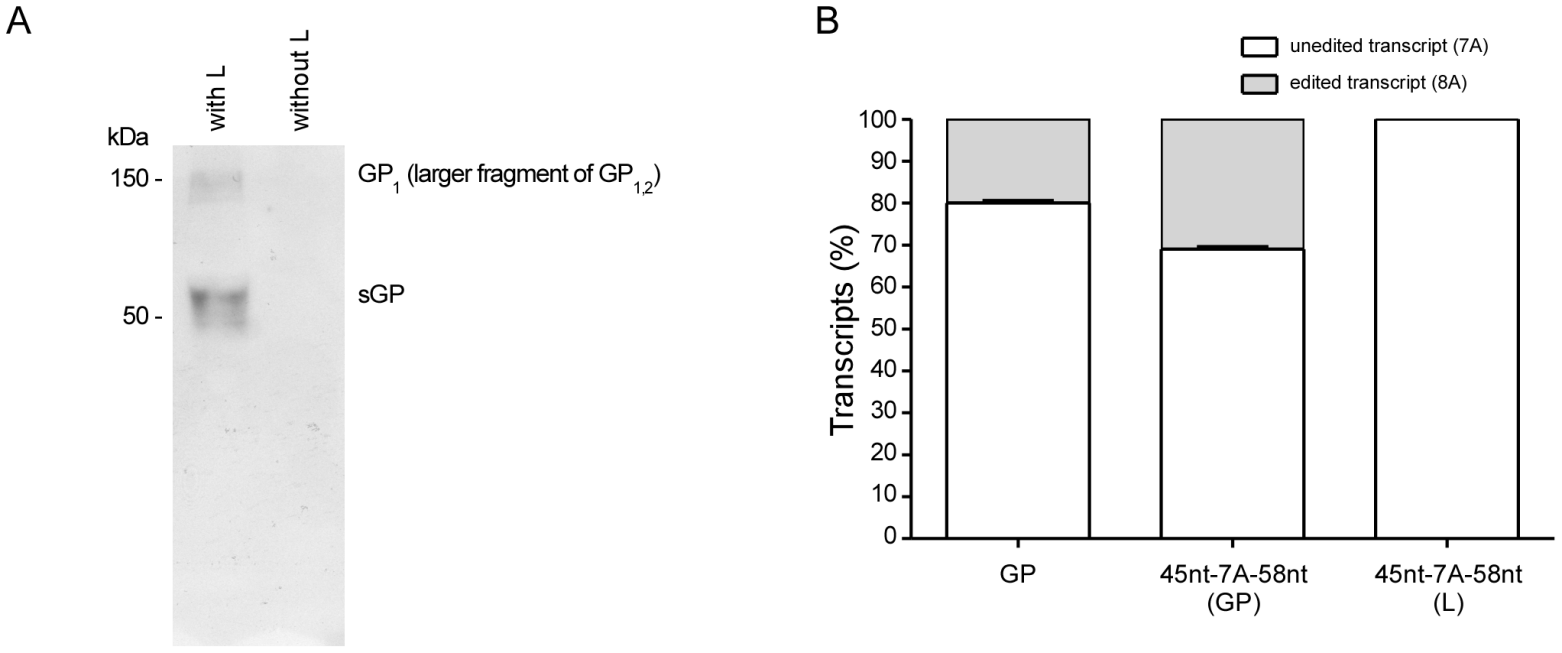
RNA editing of minigenomes. (A) GP minigenome expresses GP_1,2_ through RNA editing. Minigenome assays were performed in 293T cells using a minigenome containing the GP ORF in the presence (with L) or absence (without L; negative control) of the viral polymerase. Cells were harvested 72 hrs after transfection and lysates were subjected to SDS-PAGE under reducing conditions. GP_1_, the larger cleavage fragment of GP_1,2_, and sGP were detected using an (anti-ssGP/sGP/GP_1,2_) (1∶1,000 dilution) anti-GP antibody. (B) RNA editing is GP gene-specific. Minigenome assays were performed using minigenomes containing the full-length GP translated region (GP), a truncated version of the GP translated region spanning 110 nt around the editing site (45 nt-7A-58 nt (GP)), or a truncated version of the L gene spanning 110 nt around a putative editing site in L (45 nt-7A-58 nt (L)). Unedited (i.e. 7A) and edited (i.e. 8A) transcripts were quantified using a rapid transcript quantification assay (RTQA). Quantifications were done in triplicates from three independent minigenome rescues.

Minigenome systems model virus transcription and replication, and one particular advantage of these systems is that they allow to study the life cycle of high containment pathogens under BSL2 conditions [9]. To assess whether a minigenome system also models RNA editing, we utilized a previously established ZEBOV minigenome system [8], and replaced the reporter ORF with the GP ORF, including the editing site encoding for 7 adenosine residues, as it is found in the viral genome (7 uridine residues). This minigenome was expressed in 293T cells together with the viral RNP complex proteins (NP, VP35, L and VP30), leading to minigenome replication and transcription of mRNAs by the viral polymerase complex. Production of viral mRNAs was confirmed by detecting the major products of the GP gene, GP_1,2_ and sGP, by western blot (Figure 1A). Importantly, since GP_1,2_ is only produced after insertion of a non-templated adenosine residue into the mRNA at the GP editing site, detection of GP_1,2_ suggested that editing occurs in the context of a EBOV minigenome system. To show editing at the mRNA level, the unedited and edited transcripts encoding for sGP and GP_1,2_, respectively, were quantified using RTQA. The percentage of these major transcripts was 80 and 20%, respectively, which corresponds to the percentage of unedited vs. edited transcripts found in EBOV-infected cells, which has been reported to be 70–80% and 20–30%, respectively [3], [4] (Figure 1B, left bar).

To characterize the sequence requirements for editing, a truncated GP minigenome was generated, which contained only 110 nt of the GP translated region surrounding the editing site. RNA editing was readily detectable from this truncated GP minigenome using RTQA, and the degree of editing was comparable to the RNA editing observed with the minigenome containing the whole GP ORF. These results demonstrate that this region in the GP gene is sufficient for editing (Figure 1B, middle bar).

Interestingly, the ZEBOV L gene also contains a site encoding 7 consecutive uridine residues (genomic sense), similar to the GP gene editing site. To investigate whether RNA editing is GP gene-specific, the region surrounding the GP editing site was replaced with the corresponding region of the L gene (110 nt surrounding the potential editing site in L). The minigenome containing the truncated L region could be rescued; however, edited transcripts were undetectable by RTQA, demonstrating that transcriptional editing is GP gene specific (Figure 1B, right bar). To further confirm this finding in the context of viral infection, Vero cells were infected with ZEBOV, total RNA was extracted from infected cells, and mRNA was reverse transcribed using an oligo-dT primer. cDNA was then either subjected to RTQA, or PCR amplified using L-specific primers, followed by cloning of PCR products into the pCR 2.1-Topo TA vector for sequencing of positive clones. Both methods detected only unedited L transcripts (data not shown), further demonstrating that transcriptional editing is GP gene specific.

### The conserved editing site along with flanking sequences is required for RNA editing

**Figure 2:**
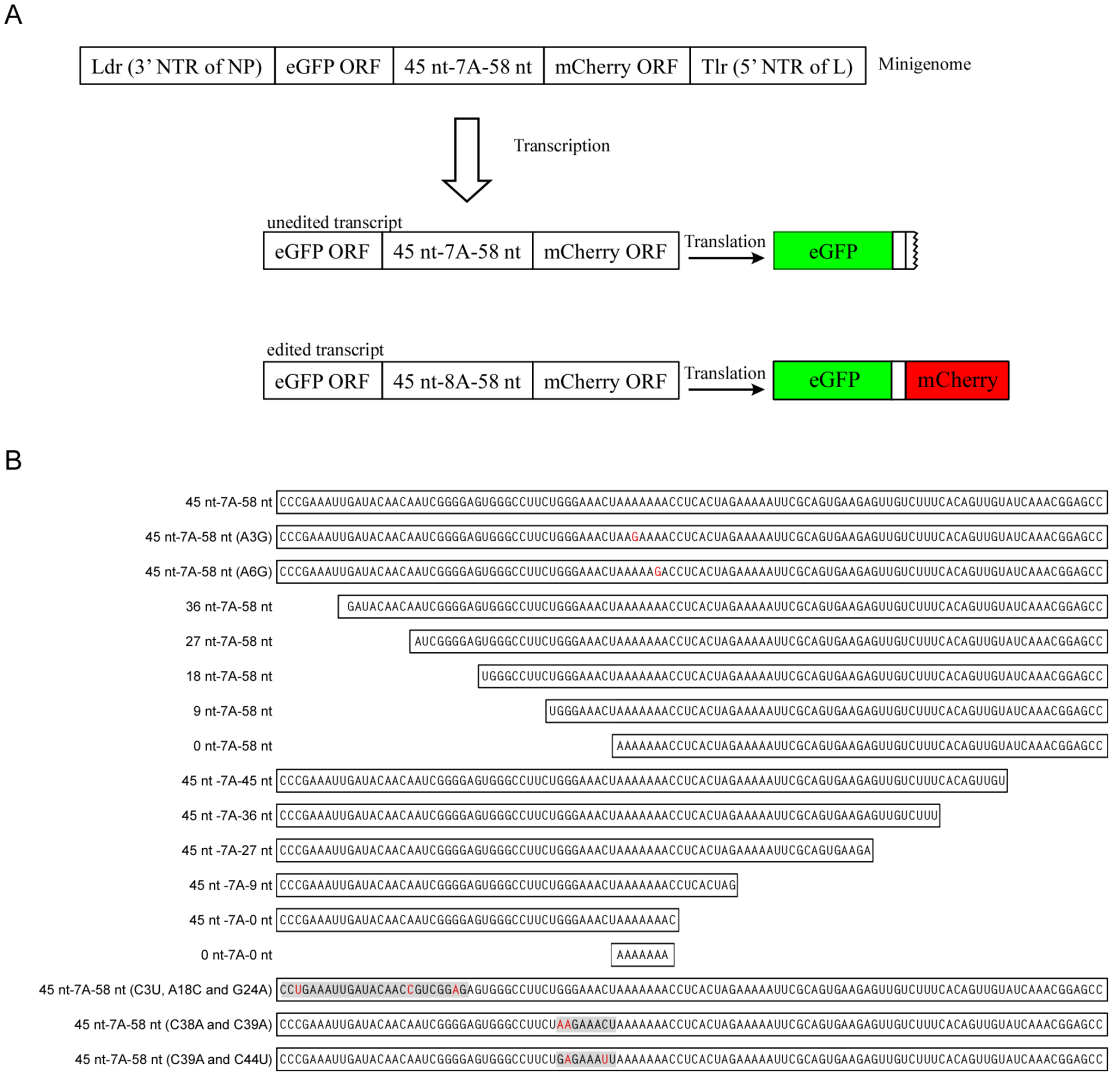
Mutations and deletions in the dual-reporter minigenome. (A) Dual reporter minigenome. A cartoon showing the structure of the minigenome, along with unedited and edited mRNA transcripts as well as the resulting reporter protein (eGFP and mCherry) expression. (B) Mutated minigenomes. Overview of the deletions and point mutations in the dual-reporter minigenome. Shown is the minigenome region corresponding to the GP translated region. Point mutations are indicated in red.

**Figure 3:**
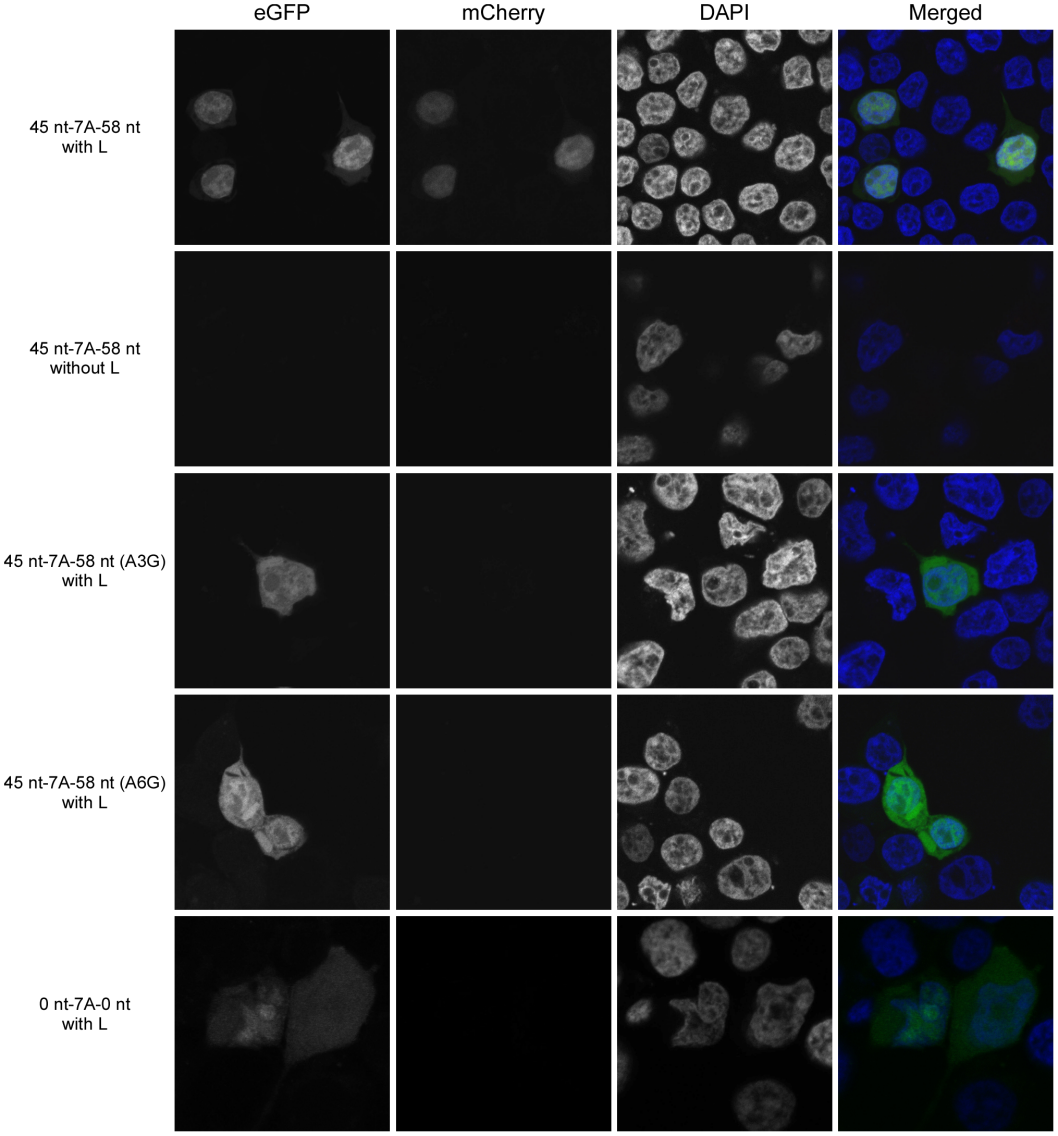
The hepta-uridine stretch in the editing site is necessary but not sufficient for RNA editing. Dual-reporter minigenome (45 nt-7A-58 nt) assays were performed in the presence (with L) or absence (without L; negative control) of the viral polymerase. The minigenomes contained either an unaltered 110 nt stretch from the GP translated region flanking the editing site, or variants with point mutations or deletions as shown in Figure 2B. Cells were analyzed for eGFP expression (from unedited and edited mRNA) and mCherry expression by fluorescence microscopy (from edited mRNA only).

To easily quantify RNA editing on the protein level, a dual-reporter cassette was developed which contained the eGFP and mCherry ORFs (Figure 2A). These two reporters were chosen because their excitation and emission spectra are far apart; therefore, no fluorescence resonance energy transfer (FRET) based interference and no background noise were observed (Figure S1). The two ORFs are connected by the 110 nt sequence flanking the GP gene editing site, with the mCherry ORF being frame shifted with respect to the eGFP ORF in such way that mCherry can be expressed only in case of RNA editing (insertion of a non-templated residue at the editing site). As expected, expression of this reporter cassette (eGFP-45 nt-7A-58 nt-mCherry) in mammalian cells resulted only in green fluorescence, whereas expression of a control cassette in which the editing site encoded 8 adenosine residues (eGFP-45 nt-8A-58 nt-mCherry) resulted in both green and red fluorescence (Figure 2A, Figure S1). This proved the feasibility of our approach to study RNA editing on protein level using a dual-reporter cassette. The dual-reporter cassette containing the editing site encoding 7 adenosine residues was cloned into a minigenome, which was then expressed in mammalian cells after transcription by the viral polymerase complex. As expected, cells exhibited both green and red fluorescence, indicating that RNA editing occurred (Figure 3). To investigate the sequence requirements for RNA editing, mutations were introduced into the primary editing site sequence by replacing the A-encoding residue at position 3 or 6 by a G-encoding residue (Figure 2B). RNA editing was completely abolished under these circumstances, as demonstrated by only eGFP expression and no evidence for mCherry expression (Figure 3; importantly, these results were consistently obtained in numerous fields of view). This indicates the importance of the primary editing site sequence for RNA editing. When both the viral sequences upstream and downstream of the editing site were deleted, but the editing site itself was kept unchanged, no editing was observed, indicating that viral sequences flanking the editing site are also required for RNA editing (Figure 3).

### Identification of the cis-acting sequences minimally required for RNA editing

**Figure 4:**
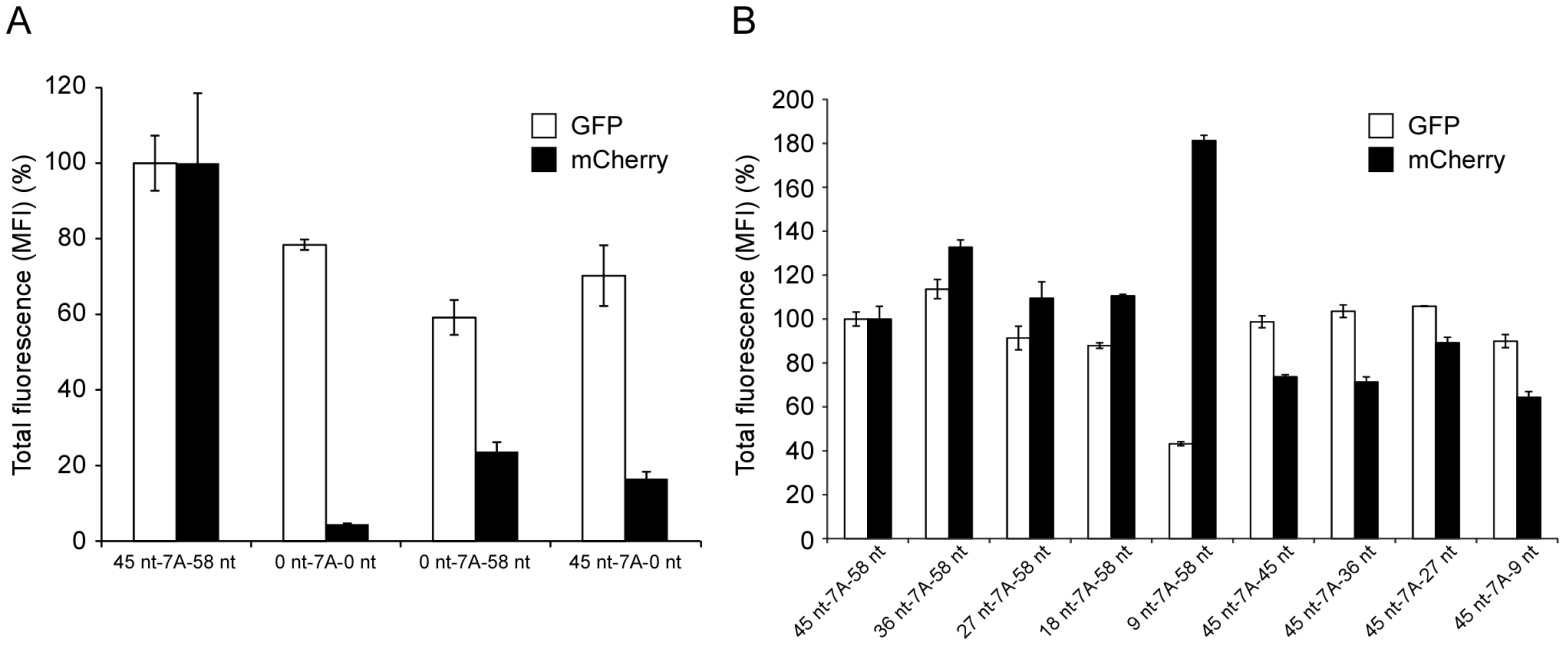
Cis-acting sequences for RNA editing reside between 9 nt upstream and 9 nt downstream of the editing site. (A) Cis-acting sequences both upstream and downstream of the editing site are important for RNA editing. Dual-reporter minigenome (45 nt-7A-58 nt) assays were performed using minigenomes containing either an unaltered 110 nt stretch from the GP translated region flanking the editing site, or variants with deletions as indicated. The mean fluorescent intensity (MFI) of eGFP (expressed from unedited and edited mRNA) and mCherry (expressed from edited mRNA only) in eGFP-positive cells were measured by flow cytometry. The relative MFI of each reporter compared to the MFI observed in context of an unaltered minigenome (45 nt-7A-58 nt) is shown. (B) Sequences 9 nt upstream and 9 nt downstream of the editing site are sufficient to support RNA editing. A series of up- and downstream deleted dual-reporter minigenomes were generated and analyzed as described in panel A. Mean and standard deviation from three independent experiments are shown.

To define which sequences surrounding the editing site are important for RNA editing, a number of mutants were generated that contained deletions in regions upstream and/or downstream of the editing site (Figure 2B). After coexpression of these minigenome mutants with the viral RNP complex proteins reporter signals from the dual-reporter minigenome were analyzed by flow cytometry, to allow for easy quantification of editing. This method was first validated by analyzing varying ratios of dual-reporter minigenomes containing an 8A editing site (surrogate for 100% editing) or dual-reporter minigenomes containing a mutated 7A editing site that did not allow for any editing to occur, resulting in a very good correlation between the input minigenomes and the measured “edited” and “unedited” mRNAs, although a very small amount of background mCherry fluorescence (3.7%) was detected in samples which did not contain mRNAs with an 8A editing site (Figure S2). When this assay was performed with a dual-reporter minigenome containing only the editing site without surrounding up- and downstream cis-acting sequences, only negligible levels of RNA editing as evidenced by reduced mCherry expression (encoded only by the edited transcript) were observed (Figure 4A, S3), similar to the results from the fluorescence microscopy analysis. Deletion of either the up- or the downstream sequences resulted in reduced RNA editing activity; although in these cases the mCherry signal was higher than that of the minigenome with just the primary editing site sequence and no surrounding viral sequences (Figure 4A). This indicates that both up- and downstream cis-acting sequences are important for RNA editing. Subsequently, the up- and downstream cis-acting sequences surrounding the editing site were consecutively deleted, as shown in Figure 2B. Coexpression of these dual-reporter minigenomes with the viral RNP complex proteins and analysis of reporter protein expression 48 hrs after transfection indicated that the region of 9 nt up- and 9 nt downstream of the editing site is sufficient to support RNA editing (Figure 4B, S3). Surprisingly, in the case of the minigenome containing only 9 nt of the upstream sequences before the editing site (9 nt-7A-58 nt), editing was actually increased as compared to the editing observed with the wild-type minigenome (45 nt-7A-58 nt).

### VP30 acts a trans-actingfactor for RNA editing

**Figure 5:**
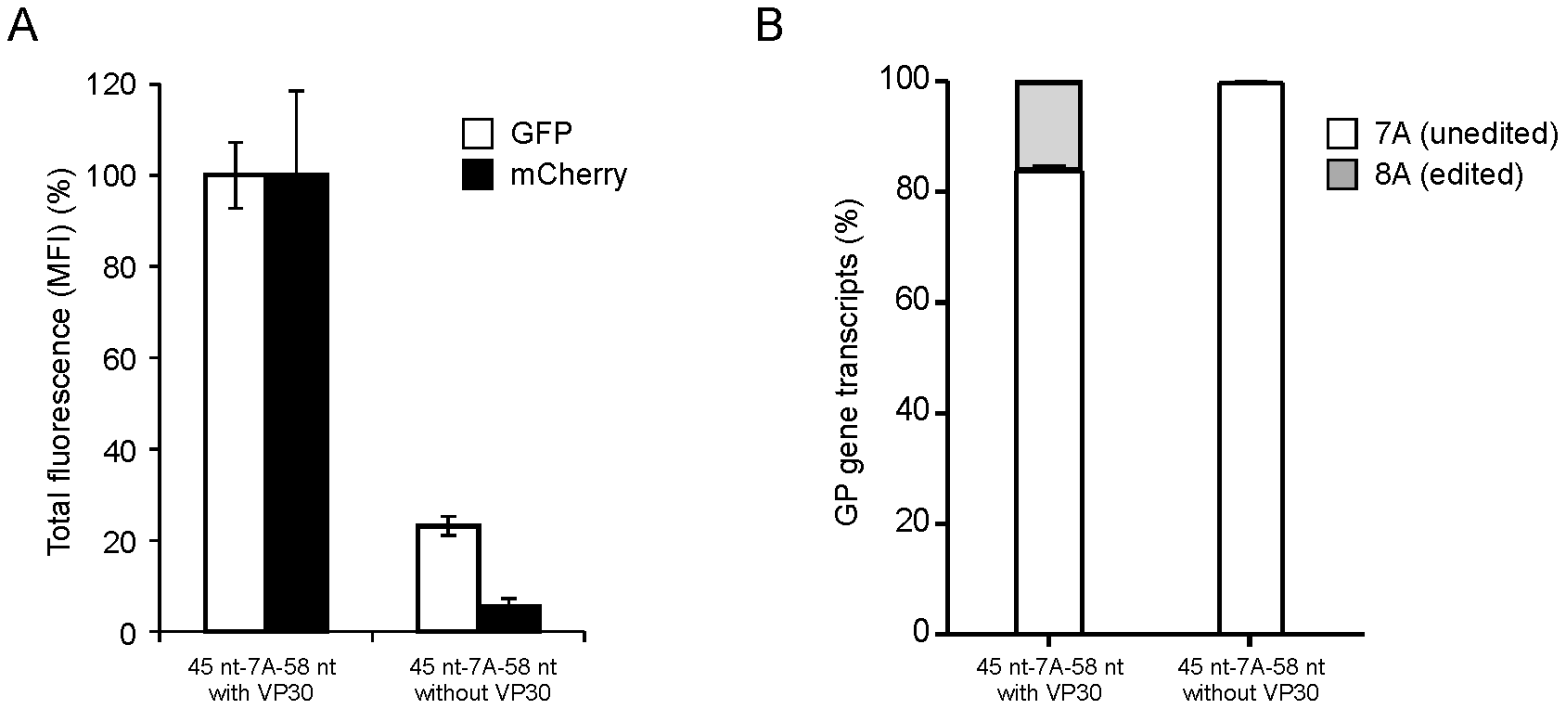
VP30 is required for RNA editing. (A) Influence of VP30 on editing as measured by reporter gene expression. Dual-reporter minigenome (45 nt-7A-58 nt) assays were performed in the presence (with VP30) or absence (without VP30) of VP30, using minigenomes containing an unaltered 110 nt stretch from the GP translated region flanking the editing site. The mean fluorescent intensity (MFI) of eGFP (expressed from unedited and edited mRNA) and mCherry (expressed from edited mRNA only) in eGFP-positive cells was measured by flow cytometry, and the intensity of each reporter in context of an unaltered minigenome (45 nt-7A-58 nt) was defined as 100%. (B) Influence of VP30 on editing as measured by transcript analysis. Minigenome assays were performed as described in panel A, and the ratio of unedited (i.e. 7A) vs. edited (i.e. 8A) transcripts was determined by RTQA. Mean and standard deviation from three independent experiments are shown.

It has been previously shown that EBOV minigenome transcription is absolutely dependent on the expression of EBOV L, NP, and VP35, but that VP30, while greatly increasing minigenome transcription, is not absolutely required for this process [8], [16]. We, therefore, investigated the role of VP30 for RNA editing using the dual reporter minigenome. As previously reported, overall reporter expression (measured by eGFP expression) was significantly reduced in the absence of VP30 (Figure 5A). However, mCherry expression showed a much more dramatic reduction in absence of VP30 (p<0.001) to levels in the range of background fluorescence (cf. Figure S2), suggesting a role of this protein for RNA editing (Figure 5A, S3). This observation was confirmed when minigenome expressing cells were analyzed by fluorescence microscopy (Figure S4). Also, when assessing the amounts of edited and unedited mRNAs using RTQA we observed edited mRNAs only in the presence of VP30, whereas in its absence no edited mRNAs were detectable, confirming the expression data (Figure 5B).

### The secondary structure of the cis-acting sequences is important for RNA editing

**Figure 6:**
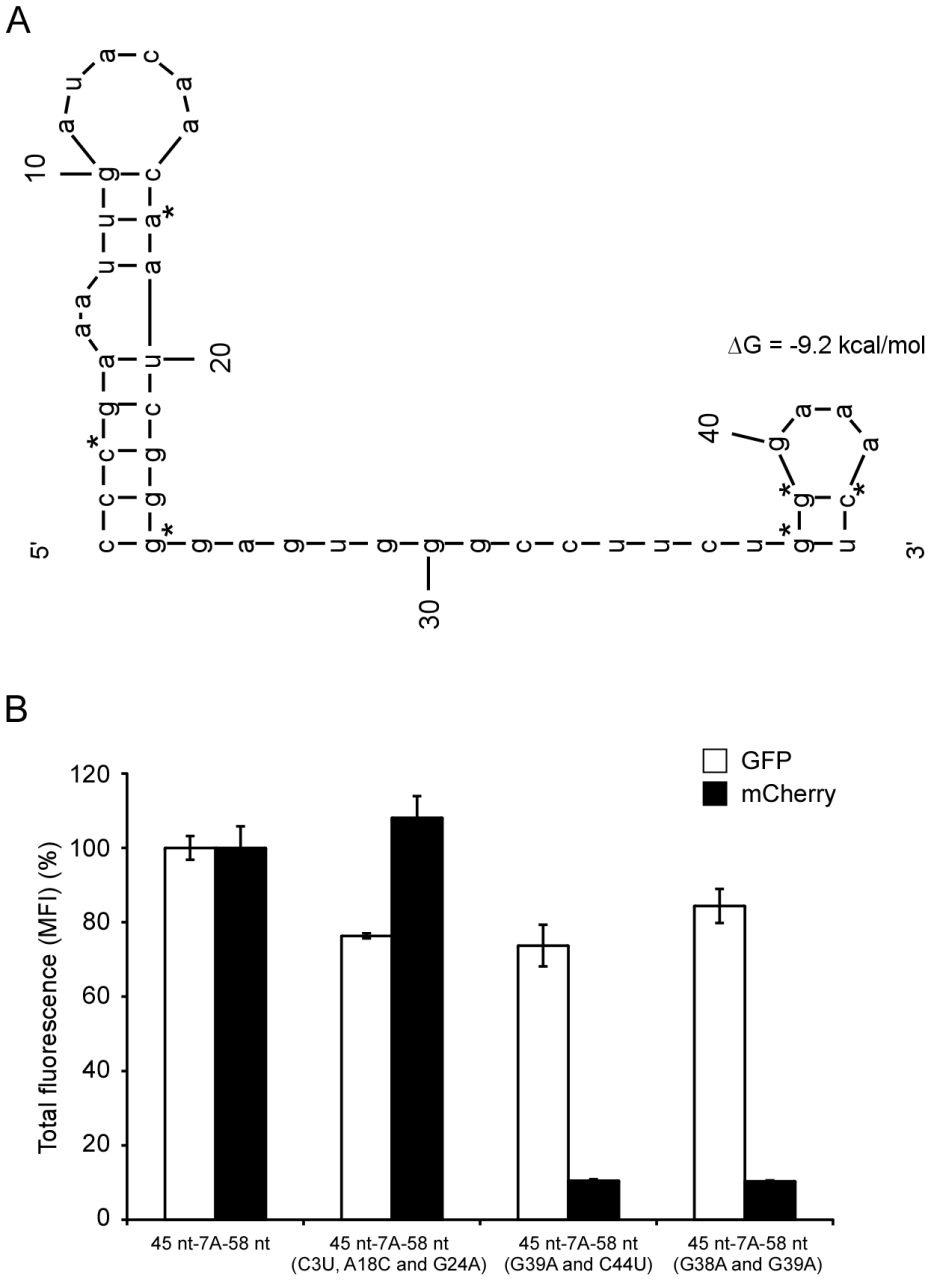
The secondary structure of the cis-acting sequence upstream of the editing site is important for RNA editing. (A) Predicted stem-loops in the 45 nt upstream of the editing site. The Mfold RNA secondary structure prediction webserver was used for secondary structure analysis of the region upstream of the editing site within the nascent mRNA. Bases that were mutated to destabilize secondary structures are marked with an asterisk. (B) The editing site proximal, but not the editing site distal stem-loop is required for editing. Mutations were introduced into the minigenome to destabilize the predicted first (45 nt-7A-58 nt (C3U, A18C and G24A)) or second (45 nt-7A-58 nt (G38A and G39A) or 45 nt-7A-58 nt (G39A and C44U)) stem-loop. Dual-reporter minigenome assays were performed using these minigenomes, and the mean fluorescent intensity (MFI) of eGFP (expressed from unedited and edited mRNA) and mCherry (expressed from edited mRNA only) in eGFP-positive cells was measured by flow cytometry. The intensity of each reporter in context of an unaltered minigenome was defined as 100%. Mean and standard deviation from three independent experiments are shown.

VP30 has been suggested to function in transcription by overcoming a secondary structure (stem-loop) prior to the NP transcription start signal [17]. We speculated that VP30 might act in a similar way during RNA editing, and, therefore, analyzed the editing site and its surrounding up- and downstream cis-acting sequences for similar secondary structures using the prediction webserver Mfold [10]. Two secondary structure models were obtained having similar ΔG values (−9.2 and −10.5 kcal/mol). Both models contain two stem-loops, each model having a stem-loop comprised of the first 24 nucleotides, but differing in the composition of the second stem-loop with one model having a stem-loop consisting of nucleotides 26 to 45 and the other consisting of nucleotides 38 to 45 (Figure 6A, S5). As we found that the upstream immediate 9 nucleotides (GGGAAACU) of the ES are critical for minigenome–driven RNA editing, that this sequence is highly conserved among all EBOV species, and that the location of GAAA resides in the loop likely forming a well-studied and stable GNRA tetraloop motif, the model containing the stem-loop formed by nucleotides 38–45 was examined further. Molecular dynamics of a tertiary structure constructed using the SYBYL program (Tripos) and based on a crystal structure of a GNRA tetraloop (PDB ID 4FNJ) was performed to determine the integrity of the predicted model. We found that the structure remains intact for the entire 18 nsec simulation with expected flexing of the three adenines in the tetraloop (Movie S1), which was not the case under similar conditions for the upstream 9 nt (UAUUUUGG) of the L gene that contains a GP gene editing site-like sequence (Movie S2). In addition, we checked for the presence of a pseudoknot for the 45 nt upstream sequence using the RNAstructure webserver [18]; however, no pseudoknot was predicted. To determine the role of the predicted stem-loops, the first putative stem-loop was destabilized by the introduction of several mutations (C3U, A18C and G24A; positions are relative to the start of the 45 nt upstream of the editing site) into the dual-reporter minigenome. Dual-reporter minigenomes were expressed in 293T cells together with the viral RNP complex proteins, and reporter protein expression were analyzed by flow cytometry. These mutations did not impair RNA editing, but rather led to a slight increase in editing, indicating that the predicted first stem-loop within the 45 nt upstream of the editing site, but outside of the region shown to be absolutely required for editing (9 nt upstream of the editing site) (Figure 4), is not required for RNA editing (Figure 6B). Subsequently, mutations were introduced into the second predicted stem-loop of the upstream cis-acting sequences (either G39A and C44U or G38A and G39A; positions are relative to the start of the 45 nt upstream of the editing site). When these minigenomes were expressed in 293T cells in the presence of the viral RNP complex proteins, mCherry expression was dramatically reduced (Figure 6B, S3), indicating the importance of the second stem-loop (formed by the 8 nt immediately upstream of the editing site) for RNA editing. In contrast, in a dual reporter minigenome with a single mutation (C44T) in this stem-loop, that does not destabilize its structure, RNA editing was not reduced, supporting the conclusion that the secondary structure rather than the primary sequence of region immediately upstream of the editing site is important for RNA editing (Figure S6).

## Discussion

The phenomenon of RNA editing of the GP mRNA of EBOV has long been described [4], [5], and been suggested to play a role in regulating GP_1,2_ expression, thereby limiting the cytotoxic effects of GP_1,2_ on host cells, and contributing to more efficient virus replication [6], [7]. Also, RNA editing has been described for other members of the order *Mononegavirales*, particularly Paramyxoviruses, for which the editing mechanism has been intensely studied [19], [20]. However, there is no information available on the mechanism of RNA editing for EBOV. Therefore, we have developed several minigenome systems that allow characterization of EBOV RNA editing both on the protein and mRNA levels, and used these systems to study the molecular details of EBOV RNA editing.

As a first step to validate this approach, the coding region of the GP gene was cloned into a minigenome system. After transcription by the viral polymerase complex, we observed expression of both GP_1,2_, which is only expressed after editing, as well as sGP (Figure 1A), similar to what is observed during virus infection. It has been reported that in certain cell lines genomic RNA editing can occur, giving rise to genomes containing an 8A editing site. However, this seems to be a much more rare event than transcriptional editing, since despite obvious increased fitness in Vero cells it takes about 4–5 passages in Vero cells for this mutation to become apparent [21], and while we cannot totally rule out that in rare cases genomic editing of minigenomes might occur, it is extremely unlikely that this was responsible for the extensive editing observed; rather, this was most likely due to transcriptional editing. Importantly, the ratio of edited vs. unedited mRNAs was similar to what has been observed during virus infection [3], [4], both when using either a minigenome containing the full-length GP ORF or a minigenome containing only a 110 nt stretch consisting of the editing site and flanking upstream and downstream sequences from the GP gene (Figure 1B). This confirmed that editing in context of a minigenome assay seems to faithfully model editing in context of viral infection. However, one point in which a minigenome assay differs from the viral life cycle is the necessity for initial transcription of the minigenome RNA from a cDNA plasmid. Our minigenome system utilized bacteriophage T7-polymerase for this step, similar to a T7-driven minigenome system that has previously been used for the characterization of paramyxoviruses RNA editing [22]. Unfortunately, T7-polymerase has previously been shown to occasionally insert non-templated adenosine residues into RNA transcribed from sequences encoding 7 adenosine residues [4], although at least parts of these observations were performed using infection with a recombinant vaccinia virus as the source for T7-polymerase, which differs from our minigenome assays. While this phenomenon suggests the possibility that analysis of editing is skewed when using T7-driven minigenome systems, several lines of evidence show that this was clearly not the case in our study. First, no editing was observed in a minigenome containing a sequence encoding 7 adenosine residues flanked by parts of the coding region of the L gene (Figure 1B). Second, T7-driven expression of a dual-reporter cassette, which contained the GP editing site along with the flanking regions of GP shown to be sufficient for viral editing, from a pTM1 expression plasmid (i.e. independent of the viral polymerase complex) did not result in any editing, as observed by the lack of functional expression of the second reporter. Third, our data show that the editing observed in the minigenome system is dependent on an EBOV protein (i.e. VP30), which was not required for the low-frequency editing by T7 previously reported.

In order to confirm and quantify RNA editing at the protein level, a dual-reporter minigenome was developed. To define the sequence requirements for RNA editing, we first investigated the role of the primary sequence of the editing site itself by introducing point-mutations. A to G mutations at positions 3 or 6 of the editing site sequence completely abolished RNA editing, indicating the importance of the primary editing site sequence (Figure 3). This is in line with previous reports, where a recombinant EBOV with identical mutations in an 8A-encoding editing site was generated and rescued that was shown to have abolished sGP production, which would have required mRNA editing [6]. Interestingly, when we analyzed RNA editing using a dual-reporter minigenome containing only the editing site without any surrounding up- and downstream viral sequences, RNA editing was dramatically reduced to almost undetectable levels (Figs. 3 and 4). This clearly indicates a requirement for surrounding cis-acting sequences for RNA editing, and explains why RNA editing does not occur during transcription of the EBOV L gene. While for EBOV such a requirement was not previously known, cis-acting sequences are known to be required for paramyxovirus P gene editing [19]. However, in contrast to paramyxovirus P gene RNA editing, which only seems to require upstream cis-acting sequences [19], further deletional mutagenesis studies showed that for EBOV RNA editing cis-acting sequences reside on both sides of the editing site (Figure 4). In particular, 9 nt upstream and 9 nt downstream of the editing site (a region spanning 25 nt in total) were identified to contain the sequences required for editing. This region is highly conserved between different EBOV species, further supporting its importance. The observation that the minigenome containing only 9 nt of the upstream sequence resulted in increased editing (Figure 4B) was surprising. This could be due to the wider sequence context of the editing site or to a more exposed stem loop (Figure 6) in this particular construct (see also discussion below).

Studying trans-acting factors for editing is complicated by the fact that all EBOV RNP proteins contribute to replication and transcription. However, among the EBOV RNP complex proteins VP30 is not absolutely required for minigenome replication and transcription, in contrast to the other RNP complex proteins, even though it greatly increases transcription [8], [16]. Therefore, it was possible to assess the impact of VP30 on editing by performing minigenome assays in absence of VP30. Interestingly, while previous publications using luciferase as a reporter gene had reported a 14-fold reduction in reporter activity, we observed only a 4-fold reduction in signal when quantifying the eGFP signal in eGFP-positive cells. This might be due to different properties of the reporter proteins. A more likely explanation, however, stems from our observation that, while the intensity of eGFP signal in positive cells was not greatly reduced, the frequency of eGFP-positive cells in absence of VP30 was much lower than that of minigenome assays performed in the presence of VP30 (data not shown). Since luciferase assays measure the sum of reporter activity from all cells, whereas our analysis was restricted to cells with reporter activity detectable, the difference in reporter activity reduction in the absence of VP30 between the different experimental systems is easily explained. More difficult to explain is the occurrence of two subset of cells, in which minigenome transcription is differentially affected by the absence of VP30; one subset of cells where transcription seems to be completely abolished (giving rise to the reduced frequency of eGFP-positive cells), and another subset where transcription is only moderately affected. A previous study has shown that replication of minigenomes is not affected by the absence of VP30, which argues against any effect of VP30 on transfection efficacy or expression level of transfected proteins and the minigenome RNA, as well as any effect of VP30 on initial transcription by the T7 polymerase or illegitimate encapsidation of naked minigenome RNAs by NP to produce RNP complexes [16]. Therefore, more likely explanations are: first, these two subsets of cells differ in the exact ratios of the other RNP complex proteins due to differences in the number of plasmids taken up by the cells during transfection, or second, these cells differ in their intracellular environment for viral transcription such as different cell cycle stages or different activation of protein kinase R (PKR), which in turn is actively influenced by EBOV RNP complex proteins [23], [24].

Surprisingly, analysis of editing both on the protein level and on transcript level showed that VP30 is clearly required for editing (Figure 5). While further studies will be required to shed light on details of the mechanism by which VP30 supports editing, it is tempting to speculate that VP30 might interact with the editing site or adjacent sequences as VP30 has been shown to directly bind viral RNA [25]. Also, it was shown that VP30 helps the polymerase complex to overcome a stem-loop-structure at the transcription start-site of the NP gene [17], which led us to the speculation that a similar structure might be involved in transcriptional editing. *In silico* analysis predicted two secondary structure models with similar ΔG obtained for the 45 nucleotides upstream of the editing site. Although the model containing the stem-loop comprised of GGGAAACU is predicted to have a slightly higher ΔG, it was chosen for further examination as it better fits several observations: first, the sequence upstream of the GGGAAACU is not required for RNA editing, second, the sequence is highly conserved among all EBOV species, and third, the sequence can potentially form a GNRA tetraloop (known to add to tertiary stability), supported by physiologically relevant molecular dynamic simulations (Movie S1). Together, these observations support the predicted model containing the GGGAAACU stem-loop. Molecular dynamics simulations of stem-loop structures corresponding to the immediate upstream 9 nt of the L gene ES, which does not invoke editing, quickly fell apart (Movie S2). The chosen model predicted two RNA stem-loops at position 1–24 and 38–45 within the 45 nt sequence upstream of the editing site (Figure 6). Destabilization of the first stem-loop had no effect on RNA editing, which was to be expected, since this region was dispensable for editing (Figure 4). In contrast, destabilization of the second stem-loop resulted in impaired RNA editing (Figure 6). This second predicted stem-loop is formed by the 8 nt immediately upstream of the editing site, and within the 9 nt upstream cis-acting sequence determined to contain regions required for RNA editing. This region along with the editing site is highly conserved, with exception of nucleotide 44 (two nucleotides upstream of the editing site) (Figure 2B). Interestingly, mutation of this nucleotide was predicted to not destabilize the stem-loop at position 38–45 and, indeed, did not reduce RNA editing, suggesting that the secondary structure rather than the primary sequence of the upstream sequence is important for editing. This is the first experimental data demonstrating a role of a secondary structure for viral RNA editing. In support of our data, a secondary structure in the editing region of the viral P gene of Simian virus 5 (SV5) has been predicted *in silico*, and suggested to contribute to RNA editing, although experimental evidence was not provided [26].

**Figure 7:**
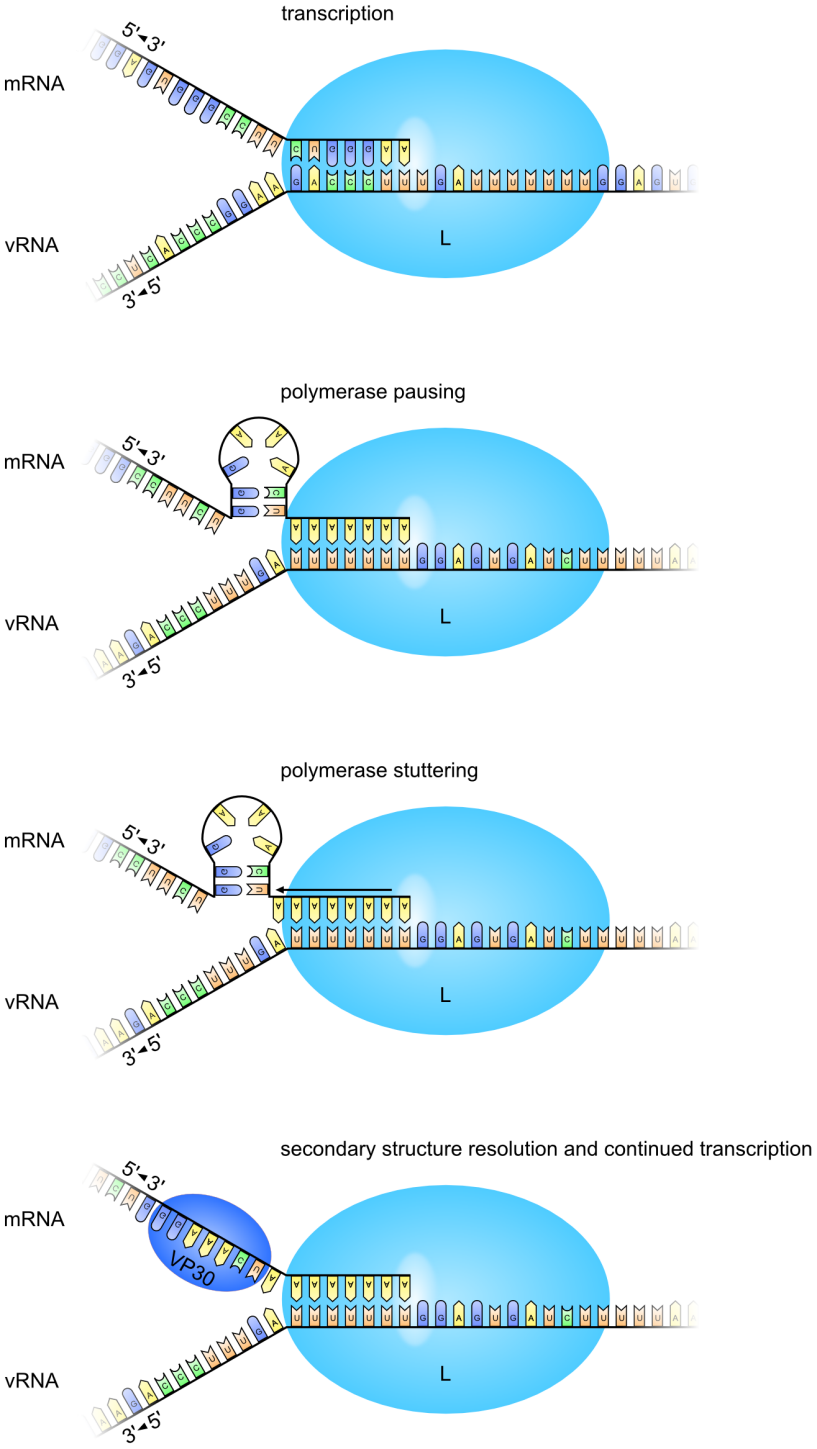
Model for EBOV RNA editing. The EBOV RNA-dependent RNA polymerase (L) transcribes vRNA into mRNA. A stem-loop structure in the mRNA directly upstream of the primary editing site causes the polymerase to pause, and enables insertion of non-templated adenosine residues due to stuttering. VP30 then resolves the stem loop, and allows continued faithful transcription.

For Paramyxoviruses, transcriptional editing has been well studied, and the current model is that upon encountering the editing site the polymerase pauses, and can then backslide on the vRNA-mRNA hybrid [19], [20]. This allows the penultimate 3′ nucleotide of the nascent mRNA to realign to the upstream residue of the template, resulting in the insertion of a pseudo-templated residue in the mRNA, sometimes referred to as polymerase stuttering [27]. We propose a similar model for ZEBOV RNA editing (Figure 7) with the difference that the sequence surrounding the editing site, in particular the stem loop structure formed by the 8 nt upstream of the editing site in the nascent mRNA, serves as a pause signal for the viral polymerase. Polymerase stuttering at the editing site, which constitutes a slippery sequence, then can occur, leading to the insertion of pseudo-templated adenosine residues into the mRNA. Subsequently, VP30 overcomes the transcription pause, similar to its function in overcoming the transcriptional pause at the beginning of the NP gene, allowing transcription to proceed. While this model is developed based on experiments with a ZEBOV minigenome, the fact that the editing site and surrounding sequences are well conserved among all EBOV species suggest that this mechanism most likely applies to all species of EBOV. It is, therefore, likely that identifying inhibitors to RNA editing might open up the potential for a novel intervention strategy to combat EBOV infections in general.

## Supporting Information

Figure S1 **Expression of the dual-reporter cassette in mammalian cells.** Dual-reporter cassettes containing either an unaltered 110 nt stretch from the GP translated region surrounding the editing site (eGFP-45 nt-7A-58 nt-mCherry; encoding for 7 adenosine residues at the editing site) or an altered version encoding for 8 adenosine residues at the editing site (eGFP-45 nt-8A-58 nt-mCherry) were cloned into a mammalian expression vector (pCAGGS) and transfected into in 293T cells. Fluorescence signals were analyzed 48 hr after transfection. As controls either eGFP or mCherry were expressed from plasmids encoding only one of these proteins.
(TIF)

Figure S2 **Validation of the FACS-based quantification of editing.** Dual-reporter minigenome (45 nt-7A-58 nt) assays were performed using minigenomes containing either an 8A editing site (surrogate for 100% editing), an 7A editing site with the 3^rd^ A mutated to a G residue, thereby abolishing editing of this minigenome (surrogate for 0% editing), or varying ratios of these two minigenomes. The mean fluorescent intensity (MFI) of eGFP (expressed from 7A and 8A containing mRNAs) and mCherry (expressed from 8A-containing mRNAs only) in eGFP-positive cells were measured by flow cytometry. The mean fluorescence intensity (MFI) of mCherry in GFP-positive cells is plotted against the relative amount of 8A minigenome for each sample.
(TIF)

Figure S3 **Normalized mCherry expression from dual-minigenome experiments.** mCherry mean fluorescence intensity from figures 4A (panel A), 4B (panel B), 5A (panel C) and 6B (panel D) was normalized to the GFP mean fluorescent intensity, providing the relative amount of editing in the respective samples.
(TIF)

Figure S4 **VP30 is a viral factor for RNA editing.** Dual-reporter minigenome (45 nt-7A-58 nt) assays were performed in the presence (with VP30) or absence (without VP30) of VP30, using minigenomes containing an unaltered 110 nt stretch from the GP translated region flanking the editing site. Cells were visualized by confocal microscopy. As a negative control, the expression plasmid encoding the viral polymerase was omitted from the transfection (without L).
(TIF)

Figure S5 **The second predicted model of the secondary structure of the cis-acting sequence upstream of the editing site with delta G = −10.50 kcal/mol).** The Mfold RNA secondary structure prediction webserver was used for secondary structure analysis of the region upstream of the editing site within the nascent mRNA.
(TIF)

Figure S6 **A single non-destabilizing mutation in the stem-loop upstream of the editing site does not reduce editing.** Dual-reporter minigenome (45 nt-7A-58 nt) assays were performed using minigenomes containing either an unaltered 110 nt stretch from the GP translated region flanking the editing site, or variants with a mutation (C44T) in the upstream of the editing site. The mean fluorescent intensity (MFI) of eGFP (expressed from unedited and edited mRNA) and mCherry (expressed from edited mRNA only) in eGFP-positive cells were measured by FACS analysis, and the intensity of each reporter in context of an unaltered minigenome (45 nt-7A-58 nt) was defined as 100%.
(TIF)

Movie S1 **Movie of an 18 nsec molecular dynamics trajectory of a GGGAAACU three-dimensional model.** The simulation includes explicit solvent (water and counterions), which are not shown. The GAAA tetraloop motif is maintained with periodic flexing of the adenines into solvent exposing their Watson-Crick faces. H-bonds = blue, GUA = green, ADE = red, URA = yellow, CYT = purple.
(MP4)

Movie S2 **Movie of an 18 nsec molecular dynamics trajectory of a UAUUUUGG three-dimensional model.** The simulation includes explicit solvent (water and counterions), which are not shown. No tetraloop motif is maintained. H-bonds = yellow, GUA = red, ADE = blue, URA = green, CYT = purple.
(MP4)

Text S1 **Detailed cloning information and primer sequences.** Provided are detailed information regarding cloning strategies and primers used for the cloning of all plasmids which were used in this study.
(DOCX)
