# Supplementary figures and images for: Ebola Virus RNA Editing Depends on the Primary Editing Site Sequence and an Upstream Secondary Structure
Source: PLoS Pathog. 2013 Oct 17;9(10):e1003677. doi: 10.1371/journal.ppat.1003677 (PMC3798607; doi:10.1371/journal.ppat.1003677)

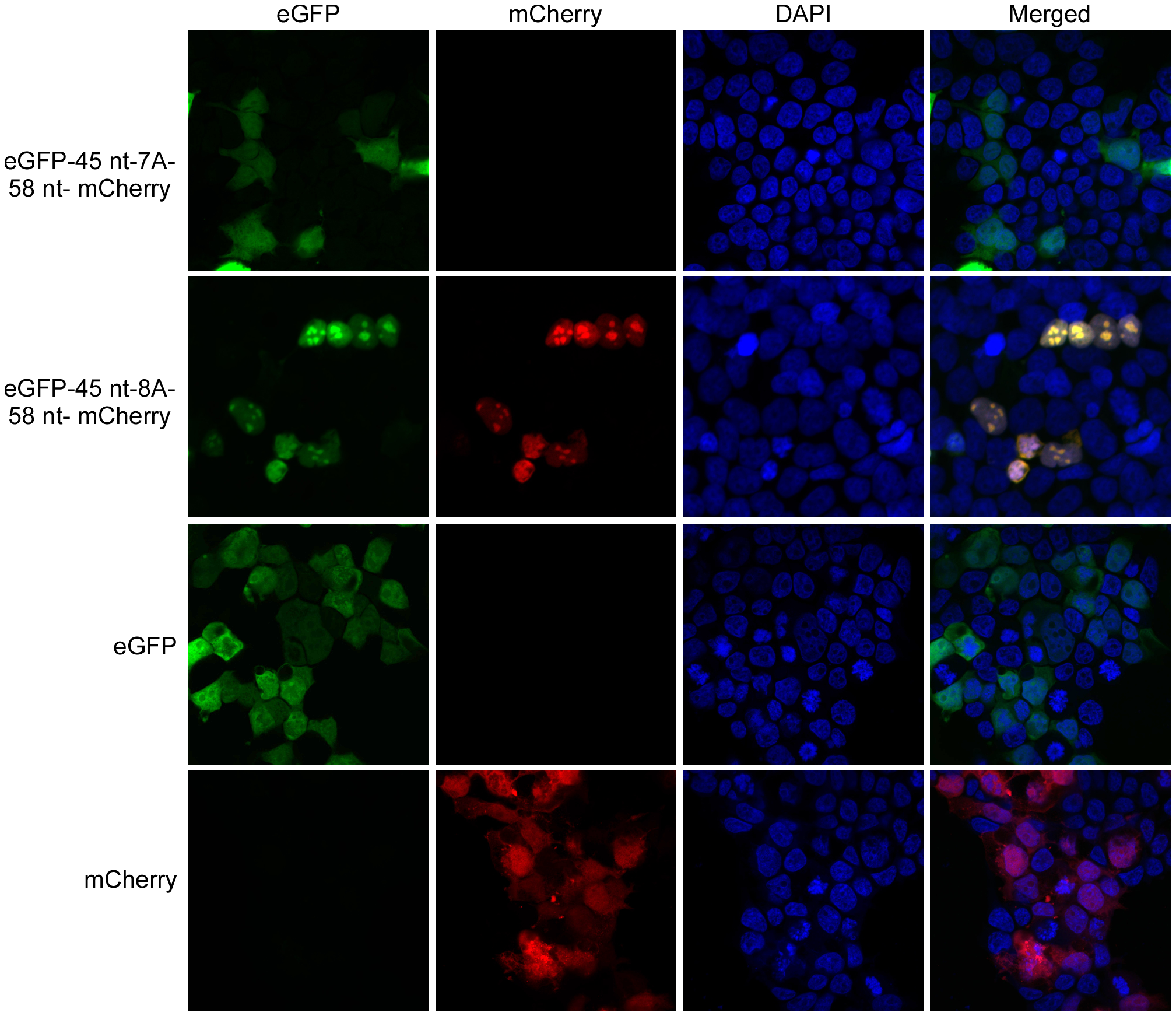

Supplement: Figure S1 — Expression of the dual-reporter cassette in mammalian cells. Dual-reporter cassettes containing either an unaltered 110 nt stretch from the GP translated region surrounding the editing site (eGFP-45 nt-7A-58 nt-mCherry; encoding for 7 adenosine residues at the editing site) or an altered version encoding for 8 adenosine residues at the editing site (eGFP-45 nt-8A-58 nt-mCherry) were cloned into a mammalian expression vector (pCAGGS) and transfected into in 293T cells. Fluorescence signals were analyzed 48 hr after transfection. As controls either eGFP or mCherry were expressed from plasmids encoding only one of these proteins. (TIF) [file ppat.1003677.s001.tif]

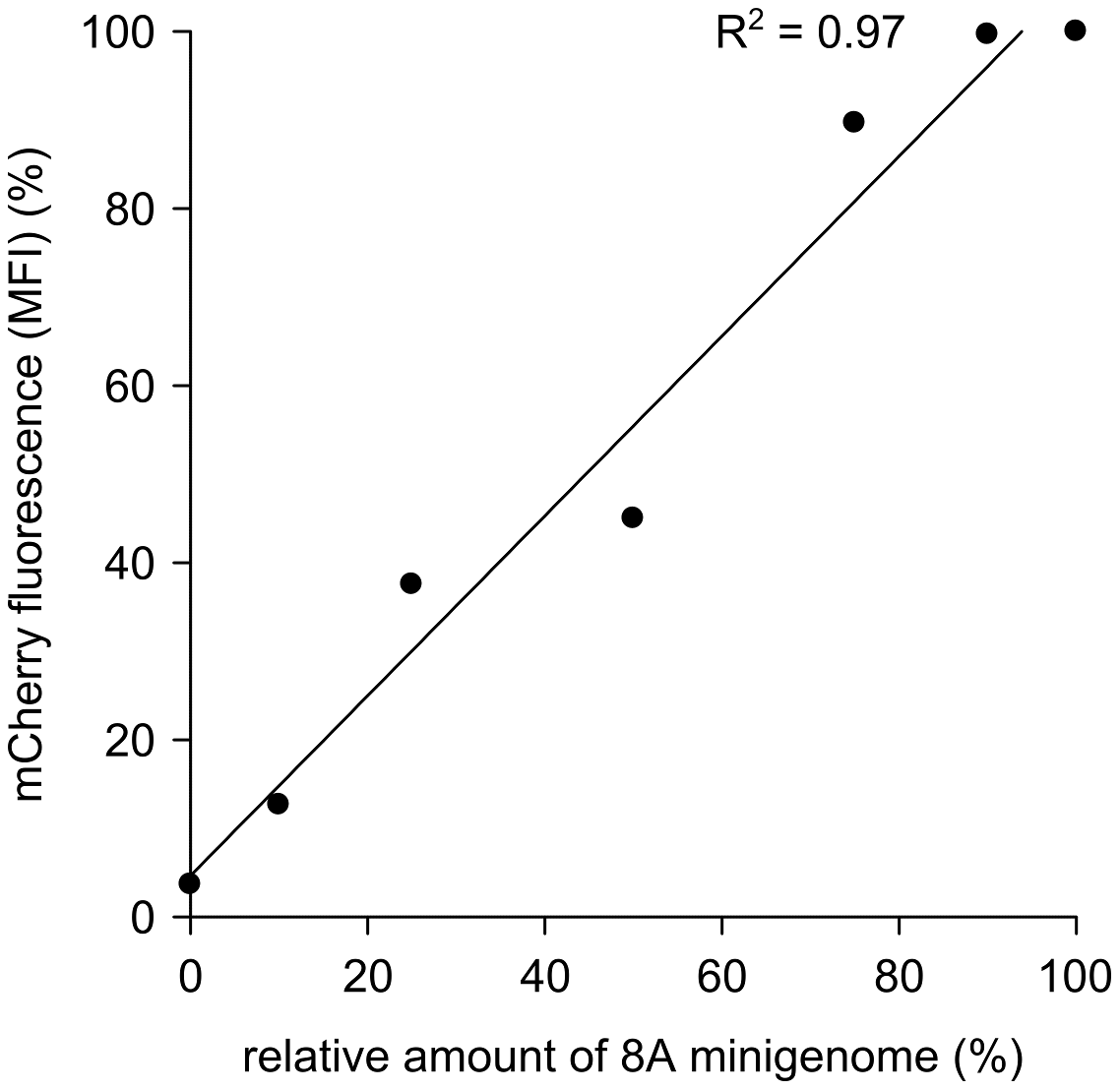

Supplement: Figure S2 — Validation of the FACS-based quantification of editing. Dual-reporter minigenome (45 nt-7A-58 nt) assays were performed using minigenomes containing either an 8A editing site (surrogate for 100% editing), an 7A editing site with the 3rd A mutated to a G residue, thereby abolishing editing of this minigenome (surrogate for 0% editing), or varying ratios of these two minigenomes. The mean fluorescent intensity (MFI) of eGFP (expressed from 7A and 8A containing mRNAs) and mCherry (expressed from 8A-containing mRNAs only) in eGFP-positive cells were measured by flow cytometry. The mean fluorescence intensity (MFI) of mCherry in GFP-positive cells is plotted against the relative amount of 8A minigenome for each sample. (TIF) [file ppat.1003677.s002.tif]

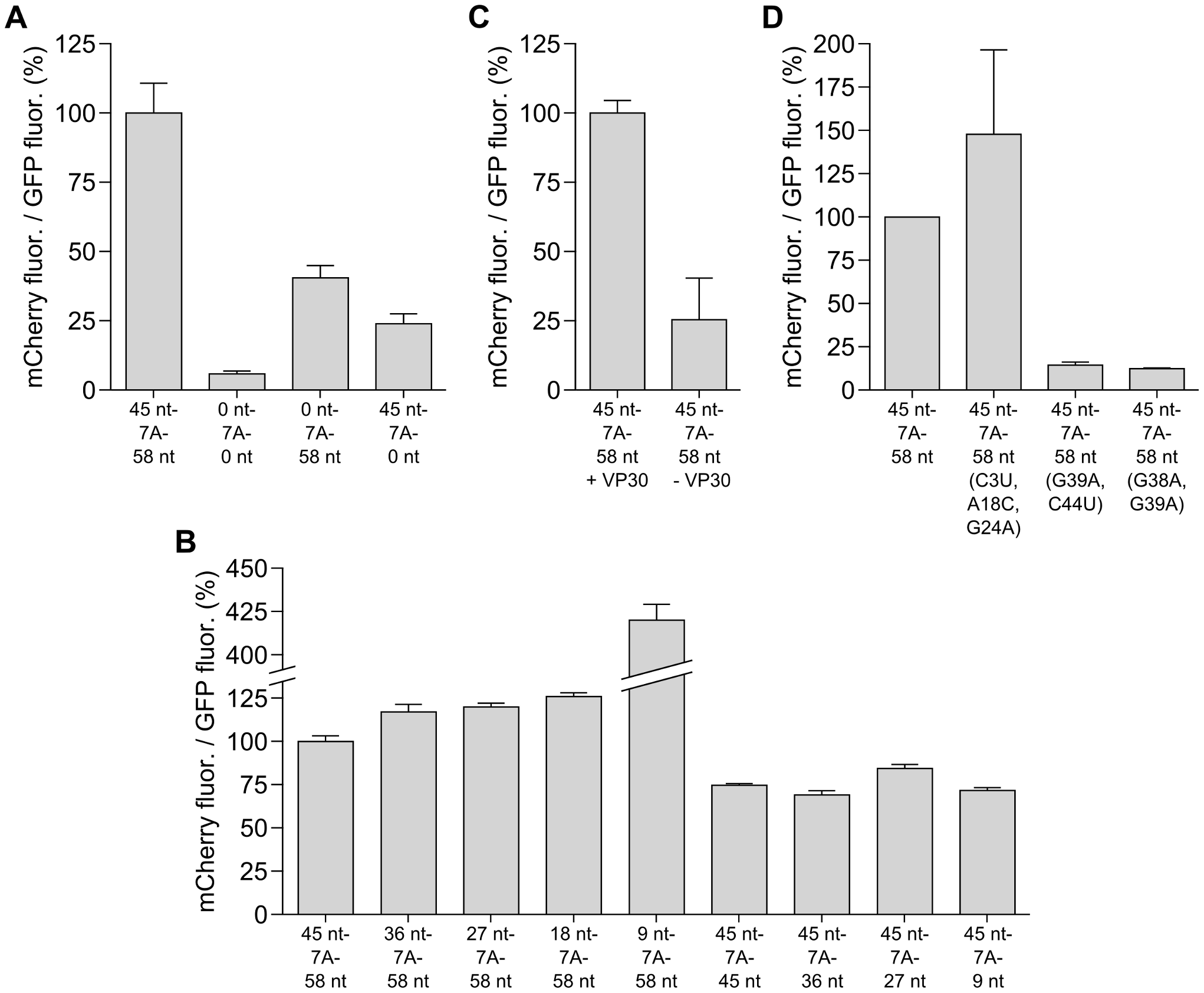

Supplement: Figure S3 — Normalized mCherry expression from dual-minigenome experiments. mCherry mean fluorescence intensity from figures 4A (panel A), 4B (panel B), 5A (panel C) and 6B (panel D) was normalized to the GFP mean fluorescent intensity, providing the relative amount of editing in the respective samples. (TIF) [file ppat.1003677.s003.tif]

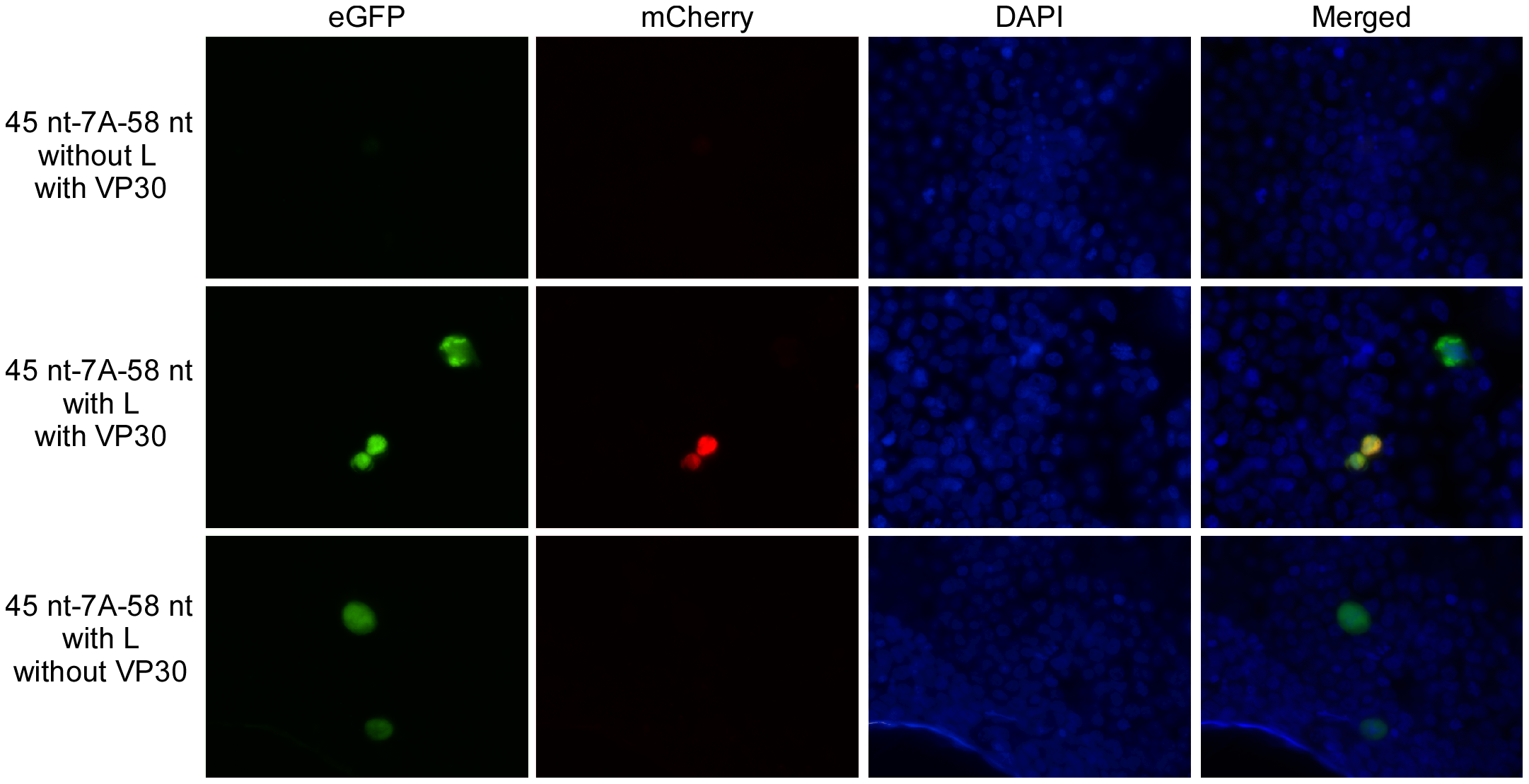

Supplement: Figure S4 — VP30 is a viral factor for RNA editing. Dual-reporter minigenome (45 nt-7A-58 nt) assays were performed in the presence (with VP30) or absence (without VP30) of VP30, using minigenomes containing an unaltered 110 nt stretch from the GP translated region flanking the editing site. Cells were visualized by confocal microscopy. As a negative control, the expression plasmid encoding the viral polymerase was omitted from the transfection (without L). (TIF) [file ppat.1003677.s004.tif]

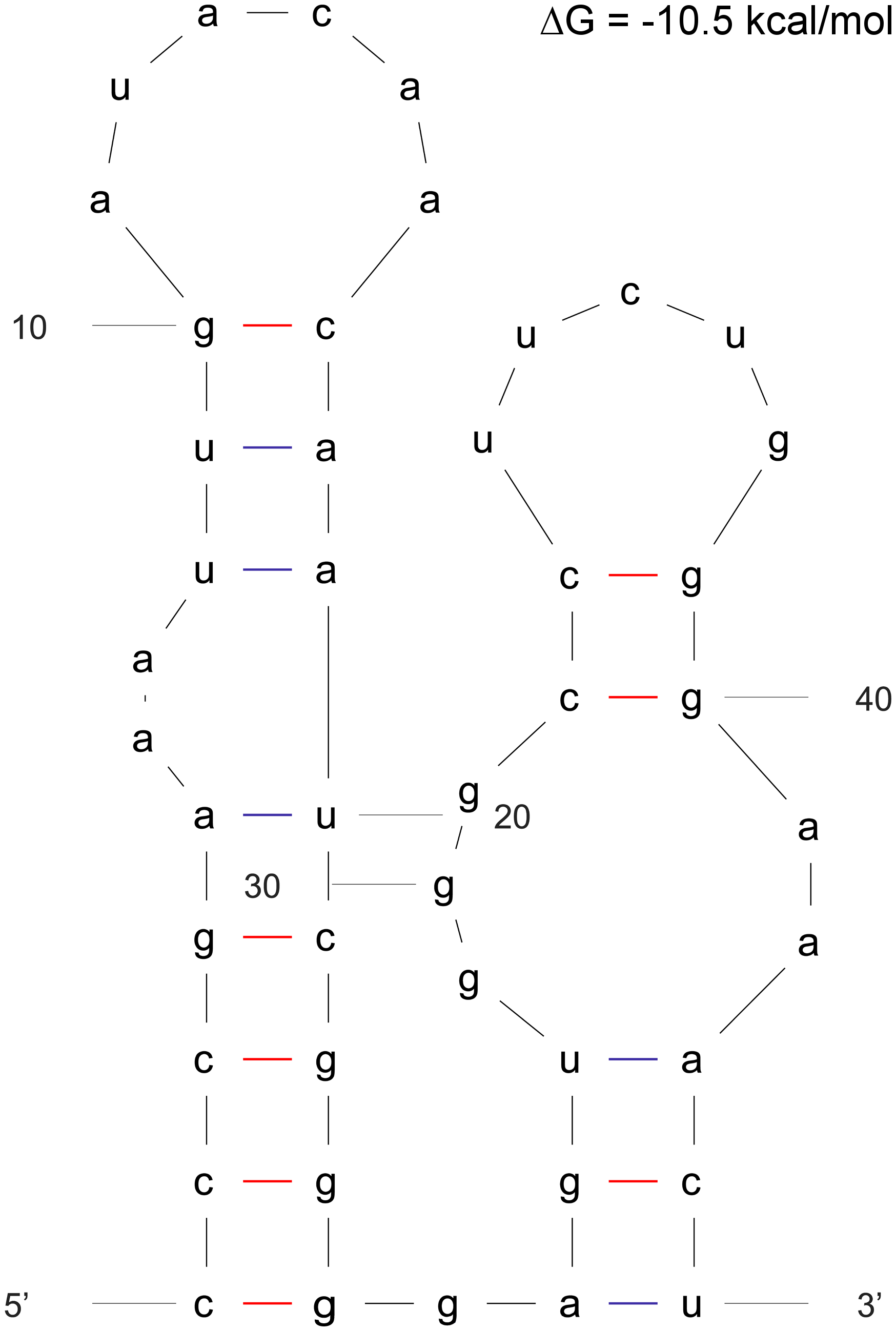

Supplement: Figure S5 — The second predicted model of the secondary structure of the cis-acting sequence upstream of the editing site with delta G = −10.50 kcal/mol). The Mfold RNA secondary structure prediction webserver was used for secondary structure analysis of the region upstream of the editing site within the nascent mRNA. (TIF) [file ppat.1003677.s005.tif]

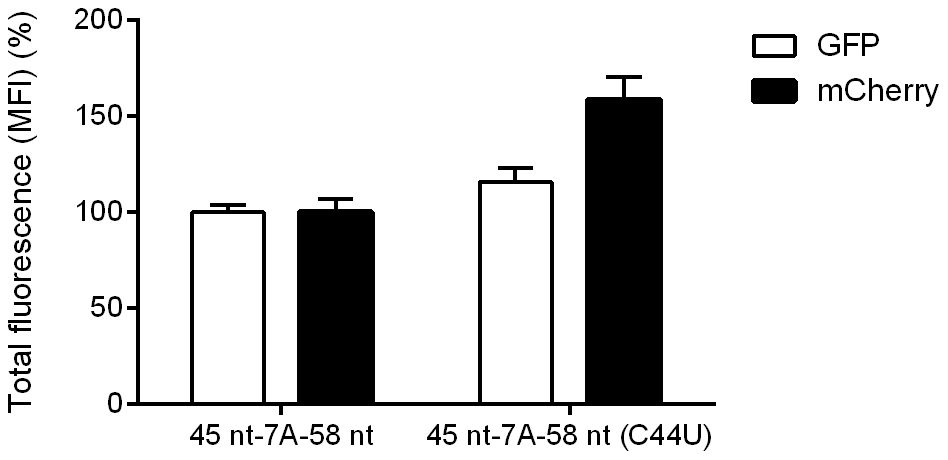

Supplement: Figure S6 — A single non-destabilizing mutation in the stem-loop upstream of the editing site does not reduce editing. Dual-reporter minigenome (45 nt-7A-58 nt) assays were performed using minigenomes containing either an unaltered 110 nt stretch from the GP translated region flanking the editing site, or variants with a mutation (C44T) in the upstream of the editing site. The mean fluorescent intensity (MFI) of eGFP (expressed from unedited and edited mRNA) and mCherry (expressed from edited mRNA only) in eGFP-positive cells were measured by FACS analysis, and the intensity of each reporter in context of an unaltered minigenome (45 nt-7A-58 nt) was defined as 100%. (TIF) [file ppat.1003677.s006.tif]
