# Supplementary material for: Ebola Virus RNA Editing Depends on the Primary Editing Site Sequence and an Upstream Secondary Structure
Source: PLoS Pathog. 2013 Oct 17;9(10):e1003677. doi: 10.1371/journal.ppat.1003677 (PMC3798607; doi:10.1371/journal.ppat.1003677)
Supplement: Text S1 — Detailed cloning information and primer sequences. Provided are detailed information regarding cloning strategies and primers used for the cloning of all plasmids which were used in this study. (DOCX) [file ppat.1003677.s009.docx]

**Supplementary Text S1:**

Minigenomes containing the full coding regions or part of the coding region of the GP and part of the coding region of the L gene were inserted into the published ZEBOV minigenome plasmid by replacing the previously used reporter gene chloramphenicol acetyltransferase (CAT) using standard cloning techniques. Briefly, two existing BsmBI sites in the minigenome plasmid backbone were deleted using site directed mutagenesis with the primers acg agg ccc ttt agt ctc gcg cgt ttc ggt g and tca ccg aaa cgc gcg aga cta aag ggc ctc g or atg cag ctc ccg gaa acg gtc aca gct tgt c and aga caa gct gtg acc gtt tcc ggg agc tgc, respectively. Then, the minigenome plasmid was PCR-amplified using primers aat cgg tac cat cgc gtc tca ttt gcg gcc gca tag tat cct gat ac and act cgg tac cgt atc gtc tca act cgg aat ttt gtg att ccg agc and religated to introduce two new BsmBI sites and to remove the CAT ORF. The GP ORF was PCR-amplified using primers act tcg tct ctg agt atg ggc gtt aca gg and ttg gcg tct cgc aaa cta aaa gac aaa ttt gc, and then digested with BsmBI, followed by ligation with the BsmBI digested minigenome plasmid. Other minigenomes were constructed using the same strategy and the following primers:

| Minigenome cassette | Forward primer | Reverse primer |
| --- | --- | --- |
| 110 nt surrounding GP editing site (45nt-7A-58nt) | attccgtctctgagtcccgaaattgatacaac | ttggcgtctcacaaaggctccgtttgatac |
| 110 nt surrounding L editing site (45nt-7A-58nt) | aacccgtctcggagtctttttaaaacctgatg | atatcgtctcgcaaaattcttgtagccgttttaagg |

The dual-reporter cassette (consisting of the GP gene editing site with surrounding sequences (45 nt-7A-58 nt) and ORFs of eGFP (without stop codon) and mCherry (without start and stop codon, and fused to a NLS sequence) up- and down-stream of the (45 nt-7A-58 nt), respectively, in a way that functional expression of mCherry would require insertion of an additional A residue into the editing site of the mRNA) was assembled in 4 steps in the subcloning vector pATX. To this end, both vector and insert were PCR amplified using the primers shown below, and ligated after BsmBI digestion.

|  | Vector | | Insert | |
| --- | --- | --- | --- | --- |
| Insert | Forward primer | Reverse primer | Forward primer | Reverse primer |
| 110 nt GP editing site | atcgtctcaaaggcggtaatacggttatcc | ttcgtctctcaggtggcacttttcggg | atcgtctcacctgcccgaaattgatacaac | ttcgtctcgccttggctccgtttgatacaac |
| eGFP-110 | acgcgtctcgcccgaaattgatacaacaatcggg | actcgtctcacaggtggcacttttcggggaaatg | atacgtctcacctgatggtgagcaagggcgag | tatcgtctcacgggcttgtacagctcgtccatg |
| eGFP-110-mcherry | atacgtctcgaaggcggtaatacggttatcc | attcgtctcgggctccgtttgatacttc | atacgtctcaagccgtgagcaagggcgagg | acgcgtctcgccttcttgtacagctcgtcc |
| eGFP-110-mCherry-NLS | attcgtctcgaaggcggtaatacggttatccacag | attcgtctcacttgtacagctcgtccatgccg | acgcgtctcgcaaggatccaaaaaagaagagaaagg | atacgtctcgccttttatctagatccggtggatcc |

Subsequently, the dual-reporter cassette was cloned into the minigenome plasmid backbone using the same strategy as outlined above, and the primers att ccg tct ctg agt atg gtg agc aag gg and acc tcg tct ctc aaa tta tct aga tcc ggt gg. For control experiments, it was cloned into pCAGGS using SacI and NheI after amplification with primers ata gag ctc atg gtg agc aag ggc g and ata gag ctc atg gtg agc aag ggc g, and into pTM1 using EcoRI and XhoI after amplification with primers ata gaa ttc atg gtg agc aag ggc gag and ata ctc gag tta tct aga tcc ggt gg. An altered version of the dual-reporter cassette containing an additional A residue in the editing site for control experiments was generated by site-directed mutagenesis using the primers aca ggg gag tgg gaa act aaa aaa aac ctc act aga aaa att cgc and aca gcg aat ttt tct agt gag gtt ttt ttt agt ttc cca ctc ccc tgt. Further point mutations and deletions were introduced into the editing site and surrounding sequences using standard cloning techniques. For upstream deletions the method described by Perez-Pinera et al. was used (Perez-Pinera, P et al., Electronic Journal of Biotechnology, 2006 Vol 9(5):604-609), and downstream deletions were done by PCR followed by BsmBI digest and religation. The following primers were used:

| Mutation/deletion | Forward primer | Reverse primer |
| --- | --- | --- |
| editing site mutation 3AG | tgggccttctgggaaactaagaaaacctcactagaaaaattcg | cgaatttttctagtgaggttttcttagtttcccagaaggccca |
| editing site mutation 6AG | tgggccttctgggaaactaaaaagacctcactagaaaaattcg | cgaatttttctagtgaggtctttttagtttcccagaaggccca |
| upstream stem-loop (1-24) mutation C3T | gcatggacgagctgtacaagcctgaaattgatacaacaatcgg | ccgattgttgtatcaatttcaggcttgtacagctcgtccatgc |
| upstream stem-loop (1-24) mutation A18C | acaagcccgaaattgatacaaccatcggggagtgggccttctgg | ccagaaggcccactccccgatggttgtatcaatttcgggcttgt |
| upstream stem-loop (1-24) mutation G24A | agcccgaaattgatacaacaatcggagagtgggccttctgggaaac | gtttcccagaaggcccactctccgattgttgtatcaatttcgggct |
| upstream stem-loop (38-45) mutation G39A&C44T | aacaatcggggagtgggccttctgagaaattaaaaaaacc | ggtttttttaatttctcagaaggcccactccccgattgtt |
| upstream stem-loop (38-45) mutation G38A & G39A | gagtgggccttctaagaaactaaaaaaacctcactag | ctagtgaggtttttttagtttcttagaaggcccactc |
| upstream stem-loop (38-45) mutation C44T | tgggccttctgggaaattaaaaaaacctcactag | ctagtgaggtttttttaatttcccagaaggccca |
| upstream 45 nt deletion | atggacgagctgtacaagaaaaaaacctcactagaaaaattcg | cgaatttttctagtgaggtttttttcttgtacagctcgtccatgc |
| upstream 36 nt deletion | atggacgagctgtacaagtgggaaactaaaaaaacc | ggtttttttagtttcccacttgtacagctcgtccat |
| upstream 27 nt deletion | atggacgagctgtacaagtgggccttctgggaaac | gtttcccagaaggcccacttgtacagctcgtccat |
| upstream 18 nt deletion | atggacgagctgtacaagatcggggagtgggccttc | gaaggcccactccccgatcttgtacagctcgtccat |
| upstream 9 nt deletion | atggacgagctgtacaaggatacaacaatcggggag | cttgtacagctcgtccatcttgtacagctcgtccat |
| downstream 58 nt deletion | attcgtctcacgtgagcaagggcgaggagg | acgcgtctcgcacgtttttttagtttcccagaagg |
| downstream 49 nt deletion | attcgtctcacgtgagcaagggcgaggagg | acgcgtctcgcacgctagtgaggtttttttagtttcc |
| downstream 31 nt deletion | attcgtctcacgtgagcaagggcgaggagg | attcgtctcgcacgtcttcactgcgaatttttctag |
| downstream 22 nt deletion | attcgtctcacgtgagcaagggcgaggagg | attcgtctcgcacgaaagacaactcttcactgcg |
| downstream 13 nt deletion | attcgtctcacgtgagcaagggcgaggagg | acgcgtctcgcacgacaactgtgaaagacaactc |
| up- and down-stream deletion using downstream deleted clone as template | acgagctgtacaagaaaaaaacgtgagcaagggcgaggagg | cctcctcgcccttgctcacgtttttttcttgtacagctcgt |

All plasmids were sequence verified.
